# iRO-PsekGCC: Identify DNA Replication Origins Based on Pseudo k-Tuple GC Composition

**DOI:** 10.3389/fgene.2019.00842

**Published:** 2019-09-18

**Authors:** Bin Liu, Shengyu Chen, Ke Yan, Fan Weng

**Affiliations:** ^1^School of Computer Science and Technology, Beijing Institute of Technology, Beijing, China; ^2^Advanced Research Institute of Multidisciplinary Science, Beijing Institute of Technology, Beijing, China; ^3^School of Informatics, Computing, and Engineering, Indiana University Bloomington, Bloomington, IN, United States; ^4^School of Computer Science and Technology, Harbin Institute of Technology, Shenzhen, China

**Keywords:** replication origin identification, pseudo *k*-tuple GC composition, random forest, web-server, DNA sequence analysis

## Abstract

**Summary:** Identification of replication origins is playing a key role in understanding the mechanism of DNA replication. This task is of great significance in DNA sequence analysis. Because of its importance, some computational approaches have been introduced. Among these predictors, the iRO-3wPseKNC predictor is the first discriminative method that is able to correctly identify the entire replication origins. For further improving its predictive performance, we proposed the Pseudo k-tuple GC Composition (PsekGCC) approach to capture the “GC asymmetry bias” of yeast species by considering both the GC skew and the sequence order effects of *k*-tuple GC Composition (*k*-GCC) in this study. Based on PseKGCC, we proposed a new predictor called iRO-PsekGCC to identify the DNA replication origins. Rigorous jackknife test on two yeast species benchmark datasets (*Saccharomyces cerevisiae*, *Pichia pastoris*) indicated that iRO-PsekGCC outperformed iRO-3wPseKNC. It can be anticipated that iRO-PsekGCC will be a useful tool for DNA replication origin identification.

**Availability and implementation:** The web-server for the iRO-PsekGCC predictor was established, and it can be accessed at http://bliulab.net/iRO-PsekGCC/.

## Introduction

In the process of the cell cycle, DNA replication is one of the most important steps (Shirahige et al., 1998). Since the DNA replication is initiated from a specific region, which is called replication origin, identifying the DNA replication origin is especially important for studying drug developments, cell life activities, genetic engineering, etc. (Méchali, 2010). Experimental methods detect the replication origins by using Chromatin immunoprecipitation (Chip) with high cost (Lubelsky et al., 2012). Therefore, researchers are seeking computational methods to efficiently predict the replication origins only based on the sequence information. Compared with non-replication origins, replication origins show uneven distribution of G (guanine) and C (cytosine) (Lobry, 1996), and the concept of “GC Skew” (Grigoriev, 1998) was proposed. Later, some computational methods incorporated these characteristics into the predictors based on the replication origins (Zhang and Zhang, 1991; Zhang and Zhang, 1994; Grigoriev, 1998; Roten et al., 2002; Thomas et al., 2007; Gao and Zhang, 2008; Luo et al., 2014; Bu et al., 2018). In order to further improve the predictive performance, the discriminative methods were proposed by using both the information of the positive and negative samples (Chen et al., 2012; Li et al., 2015; Zhang et al., 2016), and all of these methods mentioned above achieved the-state-of-the-art performance. A recent method iRO-3wPseKNC incorporated the “GC asymmetry bias” (Lobry, 1996; Grigoriev, 1998; Lubelsky et al., 2012; Li et al., 2014) into the prediction by representing the entire replication origins based on three-window-based PseKNC (3wPseKNC) (Liu et al., 2018b). Feature extraction methods are the keys for the performance improvement. In this regard, many features have been proposed, which can be easily generated by some software tools.

These existing computational methods have significantly enhanced the development of this hot area, but they all suffer from certain disadvantages or limitations, for example, as discussed above the GC Skew is an important feature of replication origins, but all the existing discriminative methods failed to directly use GC Skew to construct the predictors. Furthermore, the existing feature extraction methods cannot reflect the uneven distribution of G and C. To solve these problems, we followed the framework of iRO-3wPseKNC (Liu et al., 2018b), and proposed an improved predictor called iRO-PsekGCC for replication origin identification. iRO-PsekGCC cannot only capture the CG asymmetry bias by using *k*-tuple GC composition (or *k*-GCC), but can also incorporate the GC Skew into the concept of PseKNC (Chen et al., 2014a; Chen et al., 2014b).

## Manuscript Formatting

### Benchmark Datasets

In order to evaluate the performance of the proposed method, two recently established benchmark datasets of the *Saccharomyces cerevisiae* and *Pichia pastoris* (Liu et al., 2018b) were employed in this study, because they showed clear CG asymmetry distributions, which can be represented as:

(1) $$S_{\tau }=S_{\tau }^{+}\cup S_{\tau }^{-},\;\tau =\{\begin{array}{l} 1\text{ for }\mathrm{Saccharomyces}\;\mathrm{cerevisiae}\\ 2\text{ for }\mathrm{Pichia}\;\mathrm{pastoris} \end{array}$$

where the symbol ∪ represents the union, and $S_{-}^{+}$ represents the positive dataset containing 340 replication origins, and $S_{1}^{-}$ represents the negative dataset containing 342 non-replication origins; 305 replication origins are in positive dataset $S_{2}^{+}$, and 302 non-replication origins are in the negative dataset $S_{2}^{-}$. For both of the two benchmark datasets, the redundant samples have been removed by using CD-HIT software tool (Li and Godzik, 2006) with the most stringent cut-off threshold (80%).

### Pseudo *k*-Tuple GC Composition (PsekGCC)

One of the key steps for constructing machine-learning predictors for analyzing biological sequences is feature extraction. Following the framework of three-window-based PseKNC (3wPseKNC) (Liu et al., 2018b), we proposed a feature extraction method called “Pseudo k-tuple GC composition (PseKGCC)” to directly incorporate the CG asymmetry bias (Lobry, 1996; Grigoriev, 1998; Lubelsky et al., 2012; Li et al., 2014) and GC skew (Grigoriev, 1998) into the predictor. In the following sections, we will introduce how to represent DNA samples by using PseKGCC.

A DNA sequence D can be formulated as follow:

(2) $$D={\text{N}}_{1}{\text{N}}_{2}{\text{N}}_{3}\cdots {\text{N}}_{i}\cdots {\text{N}}_{L}\;(i=1,2,3\cdots ,L)$$

where *L* denotes the length of **D**, and

(3) $$\begin{array}{l} N_{i}\in \{A(\text{adenine}),C(\text{cytosine}),G(\text{guanine}),\\ T(\text{thymine})\},\;(i=1,2,3,\cdots ,L) \end{array}$$

which represents the *i*-th nucleobase in the sequence, and fi ∈ denotes the “member of’” in the set theory. Following the study (Liu et al., 2018b), **D** is divided into three windows by two parameters ε and δ, including front window, middle window, and rear window respectively. ε and 1 − δ denote the percentage of total nucleobases of **D** in the front window and rear window, respectively. The front window, middle window and rear window can be represented as **D**[1,η], D[η + 1,ξ], and **D**[ξ + 1, *L*], respectively, where η and ξ are defined as (Liu et al., 2018b),

(4) $$\begin{array}{ll} \{\begin{array}{l} \eta ={\mathrm{Int}}^{\text{C}}[L\times \varepsilon ]\\ \xi ={\mathrm{Int}}^{\text{C}}[L\times \delta ] \end{array}\;, & \left(0<\varepsilon <\delta <1.0\right) \end{array}$$

where the symbol Int^C^ represents the ceiling operator, which means to return the smallest integer value greater than or equal to the float number.

According to (Liu et al., 2018b), if D is formulated by the *k*-tuple nucleotide (or *k*-mer) (Liu et al., 2019b; Liu, 2017) based on the three windows strategy, it can be represented as follow:

(5) $$\begin{array}{l} D=[f_{1}^{\left(1\right)}\cdots f_{v}^{\left(1\right)}\cdots f_{4^{k}}^{\left(1\right)}f_{4^{k}+1}^{\left(2\right)}\cdots f_{4^{k}+v}^{\left(2\right)}\cdots f_{4^{k}+v}^{\left(2\right)}\cdots \\ {\;f_{2\times 4^{k}}^{\left(2\right)}f_{2\times 4^{k}+1}^{\left(3\right)}\cdots f_{2\times 4^{k}+v}^{\left(3\right)}\cdots f_{3\times 4^{k}}^{\left(3\right)}]}^{T} \end{array}$$

where in vector operations, symbol ‘T’ denotes the transformation symbol, and in the sample D, the normalized frequency values of the corresponding *k*-tuple nucleotides appearing in the front window, middle window and rear window are represented as *f* ^(1)^, *f* ^(2)^, *f* ^(3)^, respectively. The feature vector’s dimension is 3 × 4*^k^*.

This strategy was proposed to capture the patterns of “GC asymmetry bias” in yeast species genomes, and it is able to improve the predictive performance for identifying replication origins among multiple yeast species genomes. However, this approach has the following disadvantages: 1) the three windows strategy can only capture the local GC asymmetry bias of replication origins, but it cannot incorporate the GC asymmetry bias in a global fashion; 2) for large *k* values of *k*-tuple nucleotide, the dimension of the resulting feature vectors is high, which will cause high dimension disaster.

In order to overcome these disadvantages, we proposed a new composition of DNA sequence called “*k*-tuple GC composition (or *k*-GCC)” to capture the GC preference in the replication origins and their global interactions. *k*-GCC treats A (adenine) and T (thymine) as one nucleotide type represented as *. Therefore, the alphabet of *k*-GCC is

(6) $${\text{N}}_{i}\in \{\text{G}(\mathrm{guanine}),\text{ C}(\mathrm{cytosine}),*\},\;(i=1,2,3,\cdots ,L)$$

Therefore, by replacing the *k*-tuple by k-GCC, a DNA sequence D can be represented as:

(7) $$\begin{array}{l} D=[f_{1}^{\left(1\right)}\cdots f_{v}^{\left(1\right)}\cdots f_{3^{k}}^{\left(1\right)}f_{3^{k}+1}^{\left(2\right)}\cdots f_{3^{k}+v}^{\left(2\right)}\cdots f_{2\times 3^{k}}^{\left(2\right)}f_{2\times 3^{k}+1}^{\left(3\right)}\cdots \\ {\;f_{2\times 3^{k}+1}^{\left(3\right)}\cdots f_{2\times 3^{k}+v}^{\left(3\right)}\cdots f_{3\times 3^{k}}^{\left(3\right)}]}^{T} \end{array}$$

Compared with Equation 5, the *k*-GCC can efficiently reduce the dimension of the feature vector from 3 × 4*^k^* to 3 × 3*^k^* by focusing on the GC composition.

**Figure 1:**
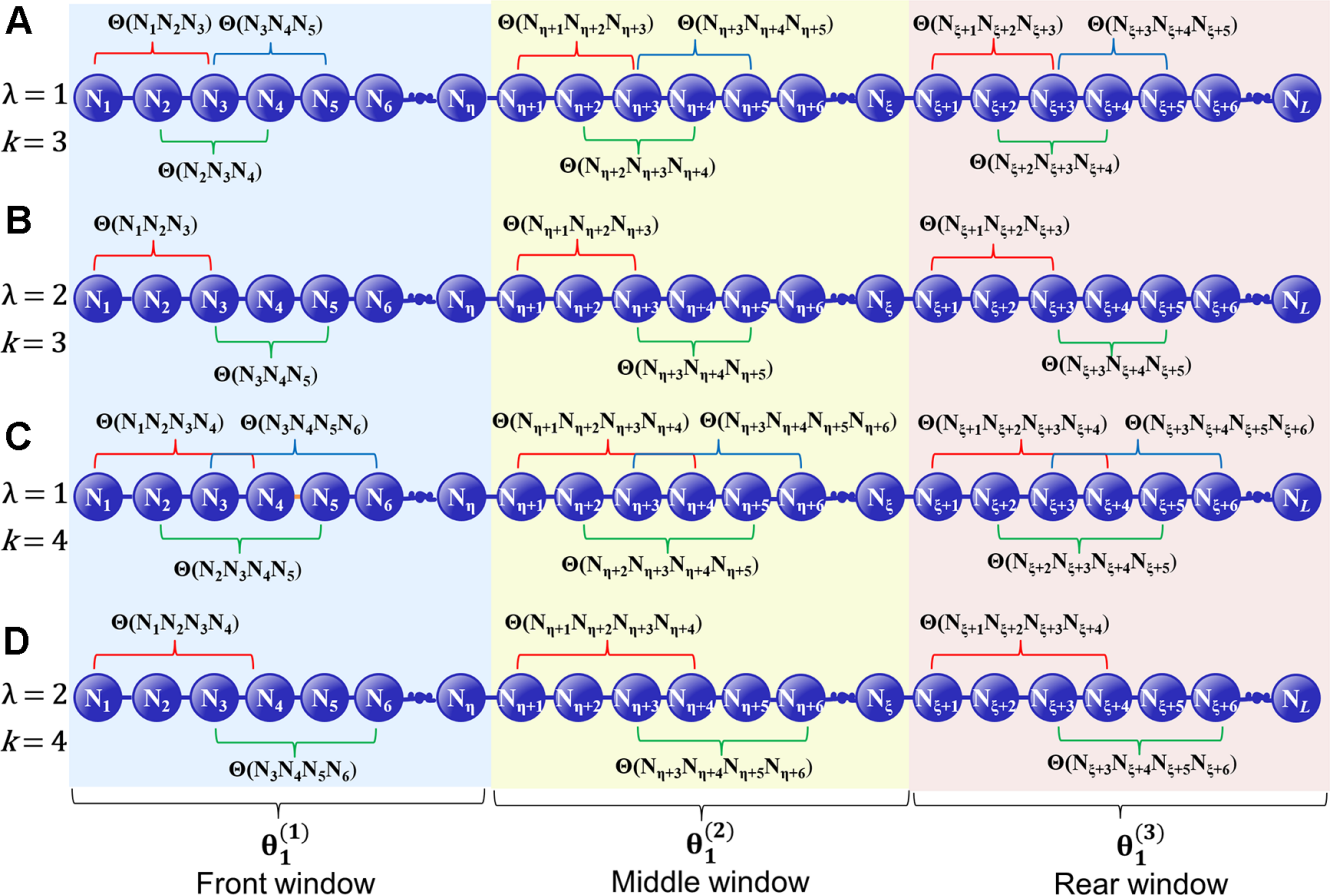
A schematic diagram to illustrate how to calculate the GC Skew in the front, middle, and rear windows along a DNA sequence. **(A)** The coupling between all the contiguous *k*-GCC (*k* = 3); **(B)** The coupling between the second most contiguous *k*-GCC (*k* = 3); **(C)** The coupling between all the contiguous *k*-GCC (*k* = 4); **(D)** The coupling between the second most contiguous *k*-GCC (*k* = 4).

The proposed Pse-KGCC incorporates both the *k*-GCC and GC skew into the framework of PseKNC (Chen et al., 2014a), which can be represented as:

(8) $$D=\begin{array}{c} {}[\phi_{1}\cdots \phi_{3^{k}}\cdots \phi_{3^{k}+\lambda }\;\phi_{3^{k}+\lambda +1}\cdots \phi_{(3^{k}+\lambda )+3^{k}}\cdots \phi_{2\times (3^{k}+\lambda )}\;\phi_{2\times (3^{k}+\lambda )+1}\\ \cdots \phi_{2\times (3^{k}+\lambda )+3^{k}}\cdots \phi_{3\times (3^{k}+\lambda )}{]}^{T} \end{array}$$

where

(9) $$\phi_{u}=\{\begin{array}{ll} \frac{f_{u}^{\left(1\right)}}{\sum_{i=1}^{3^{k}}f_{i}^{\left(1\right)}+w\sum_{j=1}^{\lambda }\theta_{j}^{\left(1\right)}} & 1\leq u\leq 3^{k}\\ \frac{w\theta_{u-3^{k}}^{\left(1\right)}}{\sum_{i=1}^{3^{k}}f_{i}^{\left(1\right)}+w\sum_{j=1}^{\lambda }\theta_{j}^{\left(1\right)}} & 3^{k}+1\leq u\leq 3^{k}+\lambda \\ \frac{f_{u}^{\left(2\right)}}{\sum_{i=3^{k}+1}^{2\times 3^{k}}f_{i}^{\left(2\right)}+w\sum_{j=1}^{\lambda }\theta_{j}^{\left(2\right)}} & 3^{k}+\lambda +1\leq u\leq 2\times 3^{k}+\lambda \\ \frac{w\theta_{u-(3^{k}+\lambda )-3^{k}}^{\left(2\right)}}{\sum_{i=3^{k}+1}^{2\times 3^{k}}f_{i}^{\left(2\right)}+w\sum_{j=1}^{\lambda }\theta_{j}^{\left(2\right)}} & 2\times 3^{k}+\lambda +1\leq u\leq 2\times 3^{k}+2\lambda \\ \frac{f_{u}^{\left(3\right)}}{\sum_{i=2\times 3^{k}+1}^{3\times 3^{k}}f_{i}^{\left(3\right)}+w\sum_{j=1}^{\lambda }\theta_{j}^{\left(3\right)}} & 2\times 3^{k}+2\lambda +1\leq u\leq 3\times 3^{k}+2\lambda \\ \frac{w\theta_{u-2\times (3^{k}+\lambda )-3^{k}}^{\left(3\right)}}{\sum_{i=2\times 3^{k}+1}^{3\times 3^{k}}f_{i}^{\left(3\right)}+w\sum_{j=1}^{\lambda }\theta_{j}^{\left(3\right)}} & 3\times 3^{k}+2\lambda +1\leq u\leq 3\times 3^{k}+3\lambda \end{array}$$

where λ denotes the highest tier correlation of the *k*-GCC nucleotides in each local window of **D**, whose the value is an integer. *w* is a float number that represents the weight factor, and the value of *w* is between 0 and 1. In the front window, the middle window and the rear window, the correlation factor of the *j*-th tier is represented as $\theta_{j}^{\left(1\right)}$, $\theta_{j}^{\left(2\right)}$, and $\theta_{j}^{\left(3\right)}$, respectively. The GC skew value of the *k*-GCC nucleotides separated by *j* nucleotides is used to represent the correlation factor of the *j*-th tier in each local window. (**Figure 1**). $\theta_{j}^{\left(1\right)}$, $\theta_{j}^{\left(2\right)}$, and $\theta_{j}^{\left(3\right)}$ can be calculated by

(10) $$128\{\begin{array}{l} \theta_{j}^{\left(1\right)}=\frac{1}{{\text{Int}}^{\text{C}}[\frac{\eta -k}{j}]+1}\overset{{\text{Int}}^{\text{C}}[\frac{\eta -k}{j}]}{\underset{i=0}{\sum }}\Theta {\text{(N}}_{i\times j+1}{\text{N}}_{i\times j+2}\cdots {\text{N}}_{i\times j+k}\text{)}\\ \theta_{j}^{\left(2\right)}=\frac{1}{{\text{Int}}^{\text{C}}[\frac{\xi -\eta -k}{j}]+1}\overset{{\text{Int}}^{\text{C}}[\frac{\xi -\eta -k}{j}]}{\underset{i=0}{\sum }}\Theta ({\text{N}}_{\eta +i\times j+1}{\text{N}}_{\eta +i\times j+2}\cdots {\text{N}}_{\eta +i\times j+k})\;\begin{array}{c} j=1,\;2,\cdots ,\lambda ;\\ \;\lambda \leq min(\eta ,\xi -\eta ,\;L-\xi ) \end{array}\\ \theta_{j}^{\left(3\right)}=\frac{1}{{\text{Int}}^{\text{C}}[\frac{L-\xi -k}{j}]+1}\overset{{\text{Int}}^{\text{C}}\left[\frac{L-\xi -k}{j}\right]}{\underset{i=0}{\sum }}\Theta \left({\text{N}}_{\xi +i\times j+1}{\text{N}}_{\xi +i\times j+2}\cdots {\text{N}}_{\xi +i\times j+k}\right) \end{array}$$

where ${\text{Int}}^{\text{C}}[\frac{\eta -k}{j}]+1$ denotes the number of the *k*-GCC in the corresponding local window, and Θ(N_*i* × *j* + 1_N_*i* × *j* + 2_ ⋯ N*_i_* × *j* + *k*) is the GC Skew (Lobry, 1996; Grigoriev, 1998; Li et al., 2014) of the *i*-th *k*-GCC in the local window, which can be calculated by

(11) $$\Theta \left({\text{N}}_{i\times j+1}{\text{N}}_{i\times j+2}\cdots {\text{N}}_{i\times j+k}\right)=\;\frac{f_{G}\left({\text{N}}_{i\times j+1}{\text{N}}_{i\times j+2}\cdots {\text{N}}_{i\times j+k}\right)-f_{C}\left({\text{N}}_{i\times j+1}{\text{N}}_{i\times j+2}\cdots {\text{N}}_{i\times j+k}\right)}{f_{G}\left({\text{N}}_{i\times j+1}{\text{N}}_{i\times j+2}\cdots {\text{N}}_{i\times j+k}\right)+f_{C}\left({\text{N}}_{i\times j+1}{\text{N}}_{i\times j+2}\cdots {\text{N}}_{i\times j+k}\right)}$$

where *f*_G_(N_*i* × *j* + 1_ N_*i* × *j* + 2_ ⋯ N*_i_* × *j* + *k*) denotes the frequency of G in the subsequence N_*i* × *j* + 1_ N_*i* × *j* + 2_ ⋯ N_*i* × *j* + *k*_ *f*_C_(N_*i* × *j* + 1_ N_*i* × *j* + *k*_ ⋯ N_*i* × *j* + *k*_) denotes the frequency of C in the subsequence N_*i* × *j* + 1_ N_*i* × *j* + 2_ ⋯ N_*i* × *j* + *k*_, reflecting the CG asymmetry bias directly. Please note that for the terminal subsequence, if its length is less than *k*, then the GC skew will be calculated by all the available nucleotide residues.

### Random Forest

Being widely used in bioinformatics (Zhao et al., 2014; Su et al., 2019), Random Forest (RF) (Ho, 1995; Barandiaran, 1998) is a machine learning classifier. Its training process can prevent overfitting (Hastie et al., 2008). The Random Forest model was implemented by calling the command line RandomForestClassifier (“max_features=’sqrt’, min_samples_leaf=1, min_samples_split=2, criterion = ‘gini’, ℱ=optimize-d value”) with the help of the Scikit-learn package (Pedregosa et al., 2011), where the values of ℱ represents the number of the trees in the forest, and it was set as 600 for both the two benchmark datasets (cf. Equation 1).

### Ensemble Learning

Previous studies (Zou et al., 2015; Liu et al., 2016a; Chen et al., 2016b; Chen et al., 2017a; Chen et al., 2017b; Liu et al., 2018a) have demonstrated that fusing a series of individual predictors by a voting strategy can improve the predictive performance. In this regard, in this study an ensemble predictors was constructed by fusing 10 top performing individual predictors constructed by different parameter combinations of PseKGCC (see **Supplementary Information S1**), which can be represented as (Liu et al., 2016a):

(12) $$\mathbb{R}F^{\text{E}}=\text{RF(1) }\forall \text{ RF(2) }\forall \;\cdots \forall \text{ RF(}i\text{) }\forall \cdots \forall \text{ RF(10)}=\forall_{i=1}^{10}\text{RF}(i)$$

where $\mathbb{R}F^{\text{E}}$ represents the ensemble classifier, ∀ represents the fusing operator, and RF(*i*) represents the basic Random Forest predictor.

**Figure 2:**
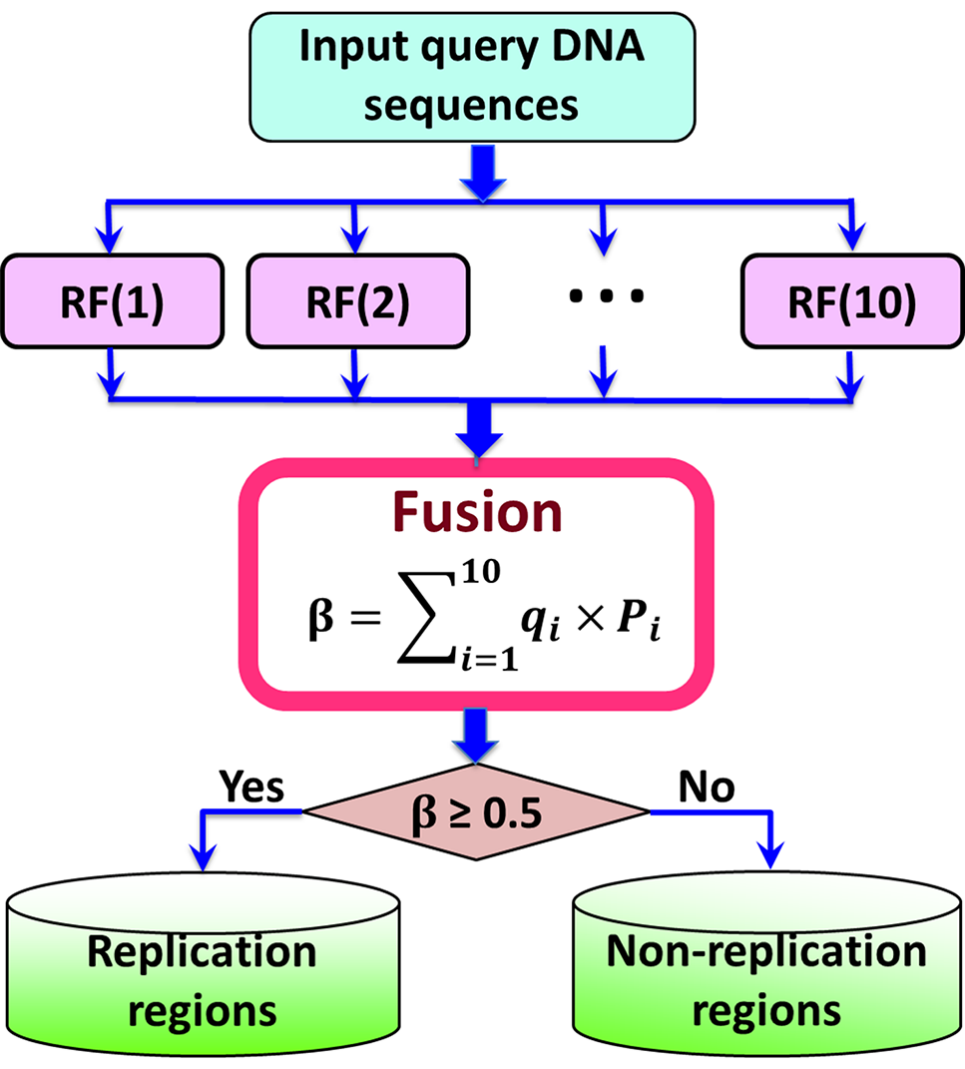
A flowchart illustration to show how the iRO-PseKGCC predictor works.

The ensemble predictor is constructed based on the fusion score ß of the probabilities predicted by the 10 basic predictors, which can be calculated by

(13) $$\text{ß}=\;\sum_{i=1}^{10}q_{i}P_{i}$$

where *q* *_i_* is the weight of the *i*-th basic RF predictor, which was optimized by the genetic algorithm (Mitchell, 1998), and their values were listed in **Supplementary Information S1**. If the value of ß is higher than 0.5, it is a replication origin, otherwise, it is a non-replication origin. The flowchart of the iRO-PseKGCC is illuminated in **Figure 2**.

### Cross Validation

Three widely used cross-validation strategies include: i) independent test, ii) K-fold cross validation, and iii) jackknife test. Among these methods, only the jackknife test can achieve the unique results for the same benchmark dataset. Therefore, in this study, the jackknife test was employed to give the final predictive results. However, considering its high computational cost, during the parameter optimization process, the 5-fold cross-validation was used to reduce the computational cost (see *Optimize Parameters* section).

### Evaluation Method of Performance

To evaluate the quality of the classifier for prediction of the replication origins, the four metrics are used (Feng et al., 2013; Chen et al., 2016c; Chen et al., 2019): i) the sensitivity, Sn, ii) the specificity, Sp, iii) the overall accuracy of the predictive results, Acc, iv) the Mathew’s correlation coefficient, MCC, and v) Arear under ROC Curve, AUC (Chen et al., 2016a), defined as:

(14) $$\{\begin{array}{ll} \text{Sn}=1-\frac{N_{-}^{+}}{N^{+}} & 0\leq \text{Sn}\leq \text{1}\\ \text{Sp}=1-\frac{N_{+}^{-}}{N^{-}} & 0\leq \text{Sp}\leq \text{1}\\ \text{Acc}=1-\frac{N_{-}^{+}+N_{+}^{-}}{N^{+}+N^{-}} & 0\leq \text{ACC}\leq \text{1}\\ \text{MCC=}\frac{1-\left(\frac{N_{-}^{+}+N_{+}^{-}}{N^{+}+N^{-}}\right)}{\sqrt{\left(1+\frac{N_{+}^{-}+N_{-}^{+}}{N^{+}}\right)\left(1+\frac{N_{-}^{+}+N_{+}^{-}}{N^{-}}\right)}} & -1\leq \text{MCC}\leq \text{1}\\ AUC & \text{Arear under ROC Curve} \end{array}$$

where *N* ^+^ denotes the number of all the positive samples (replication origins), *N* ^−^ denotes the number of all the negative samples (non-replication origins), $N_{-}^{+}$ denotes the number of the positive samples (replication origins) incorrectly predicted as the negative samples (non-replication origins), $N_{+}^{-}$ denotes the number of the negative samples (non-replication origins) incorrectly predicted as the positive samples (replication origins). More information of these performance measures can refer to Liu et al. (2016b).

## Results and Discussion

### Optimize Parameters

There are five parameters in PseKGCC according to Equations 4–9. These parameters were optimized by the following equations:

(15) $$\{\begin{array}{ll} 0.15\leq \varepsilon \leq 0.5, & \text{with step}△\varepsilon =0.05\\ 0.5<\delta \leq 0.85,\; & \text{with step}△\delta =0.05\\ 3\leq k\leq 7, & \;\text{with step}△k=1\\ 1\leq \lambda \leq 10, & \text{with step}△\lambda =3\\ 0.1\leq w\leq 1,\; & \text{with step}△w=0.1 \end{array}$$

The fivefold cross-validation was employed to search the optimal parameters by gridding method so as to reduce the time consumption, and the predictive results of the top 10 performing predictors, and their optimized parameters were listed in **Supplementary Information S1**.

### Comparison With Other Methods

To the best knowledge of ours, iRO-3wPseKNC (Liu et al., 2018b) is the only existing predictor that is able to predict the entire replication origins. All the other predictors can only predict the fragments of replication origins. Therefore, the performance of the proposed iRO-PseKGCC was compared with iRO-3wPseKNC on the two benchmark datasets, and the results were listed in **Table 1**, from which we can see that iRO-PseKGCC obviously outperformed iRO-3wPseKNC in terms of the five performance measures (cf. Equation 14), indicating that the proposed PseKGCC feature is able to capture the GC asymmetry bias, and incorporate the GC skew into the predictor. Therefore, iRO-PseKGCC is an efficient approach for improving the predictive performance.

**Table 1:** The results of the iRO-PseKGCC Predictor and comparison with iRO-PseKGCC on the two benchmark datasets (cf. Equation 1) obtained by using jackknife test.

Species	Method	Acc(%)	MCC	Sn(%)	Sp(%)	AUC
*Saccharomyces cerevisiae* $S_{1}$	iRO-PseKGCC^a^	76.46	0.5298	73.90	78.13	0.8129
	iRO-3wPseKNC^b^	72.95	0.4594	70.67	75.22	0.8084
*Pichia pastoris* $S_{2}$	iRO-PseKGCC^a^	74.22	0.4844	74.51	73.93	0.8002
	iRO-3wPseKNC^c^	71.10	0.4222	69.93	72.28	0.7962

a The parameters are listed in **Supplementary Information S1**.

b The predictor reported in (Liu et al., 2018b) with parameter ε = 0.25, δ = 0.85, k = 5, λ= 6, w = 0.3, and ℱ = 700.

c The predictor reported in (Liu et al., 2018b) with parameter ε = 0.15, δ = 0.55, k = 4, λ = 9, w = 0.3, and ℱ = 800.

### Feature Analysis

Random forest is a combination classifier model composed of decision tree classifiers. During the process of constructing each tree by the “Bootstrap” method (Efron, 1992), samples not extracted for training the corresponding tree can be used to make “Out Of Bag” (OOB) error estimate (Breiman, 1996) to evaluate the generalization performance of a predictor. Based on the OOB error, the Mean Decrease Accuracy (MDA) (Jiang et al., 2007) can be used to estimate the importance of the features. The details of the process are (Jiang et al., 2007): 1) When training a Random Forest model, using the OOB samples to test the accuracy of each tree in the model; 2) Randomly disturb the value of the feature variable *v* in the OOB samples, and retest the accuracy of each tree; 3) Calculate the mean value of the decreasing accuracy between the two tests in all decision trees in the Random Forest model. The MDA value can reflect the importance of the corresponding feature.

As shown in previous studies (Liu and Zhu, 2019; Liu et al. 2019a), feature analysis is critical for exploring the characteristics of the predictors. To explore the reason why the proposed predictor iRO-PseKGCC works so well, we analyzed the features of the two top performing iRO-PseKGCC predictors (see **Supplementary Information S1**) on the two benchmark datasets (cf. Equation 1) by MDA approach, and the results are listed in the **Table 2**, from which we can see that: 1) for both the two RF-based predictors, their most important features are the “***” and “*****,” indicating the importance of the *k*-GCC; 2) The global sequence order effects measured by different λ values and GC skew values contribute to the performance improvement; 3) Features in certain local window show more discriminative powers than those in other windows, for examples, for *Pichia pastoris*, all the top 10 most important features are in the middle window, which is consistent with the previous observations that the nucleobase composition distribution is uneven along the replication origins (Lobry, 1996; Grigoriev, 1998; Frank and Lobry, 1999; Tillier and Collins, 2000; Liu et al., 2018b).

**Table 2:** The top 10 most important features of the top two performing RF-based predictors on the two benchmark datasets (cf. Equation 1).

Rank	Saccharomyces cerevisiae			Pichia pastoris		
	Feature	Window	MDA (%)	Feature	Window Index	MDA (%)
1	***	Rear window	20.49	*****	Middle window	15.89
2	***	Middle window	19.62	****G	Middle window	5.69
3	*GG	Rear window	9.04	G****	Middle window	5.38
4	GG*	Rear window	8.35	*C***	Middle window	5.23
5	*GG	Middle window	8.26	*G***	Middle window	5.14
6	λ = 1	Rear window	7.67	*CGCG	Middle window	3.99
7	GG*	Middle window	7.45	****C	Middle window	3.94
8	CC*	Middle window	7.31	***G*	Middle window	3.77
9	G*G	Rear window	6.64	*C*GG	Middle window	3.47
10	λ = 2	Rear window	6.12	C**G*	Middle window	3.40

### Web Server and User Guide

Web-servers are important for the researchers to implement the corresponding computational predictors. In this regard, for the user’s convenience, we established a web-server named “iRO-PseKGCC.” For users’ convenience, a detailed user guide explaining how to use the web-server is given.

**Figure 3:**
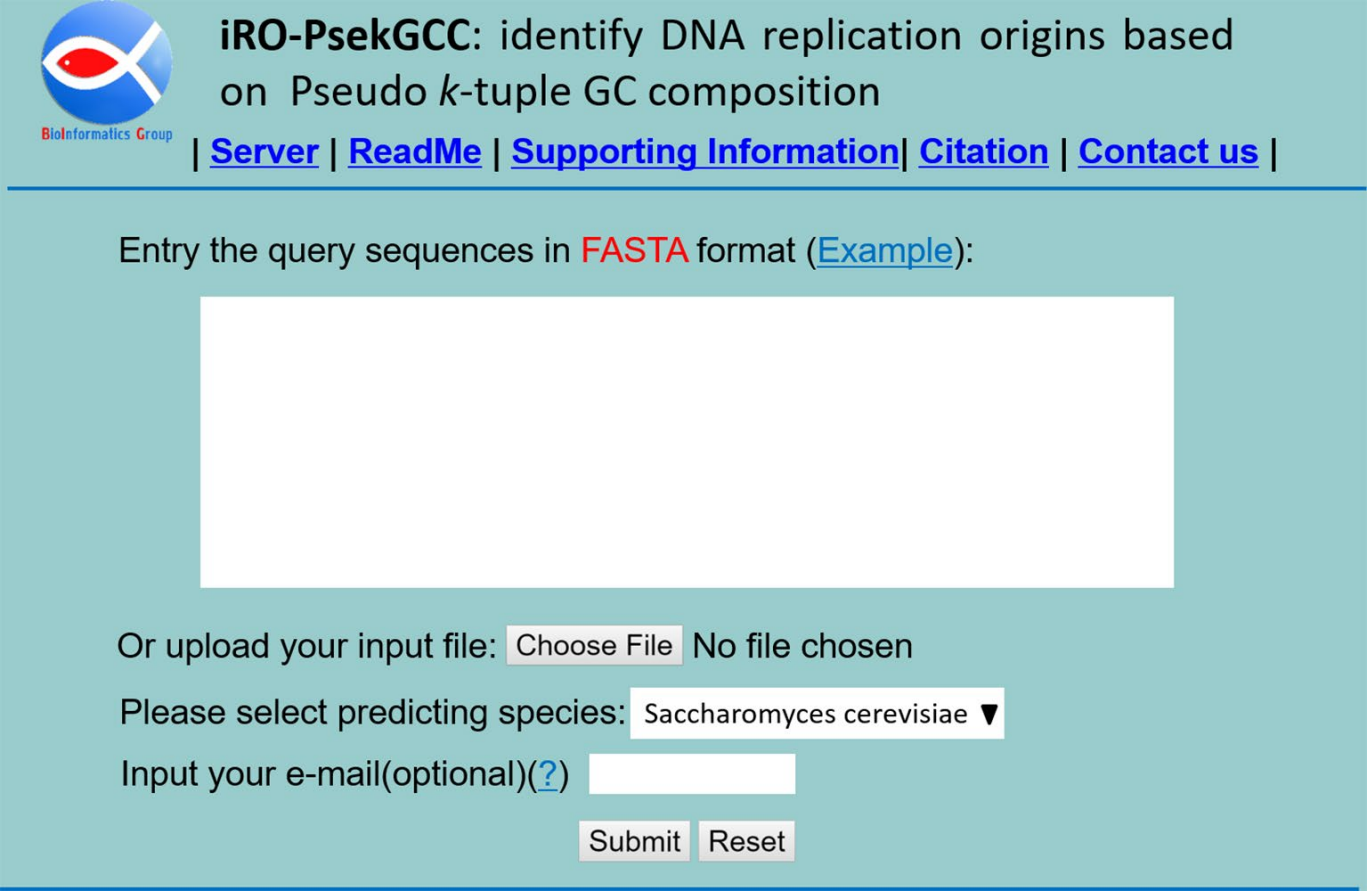
A semi-screen shot to show the homepage of the web-server iRO-PseKGCC, which can be accessed at http://bliulab.net/iRO-PsekGCC/.

**Step 1**. Click on the web sites address http://bliulab.net/iRO-PsekGCC/ to open the web-server, then the main pages on the website as shown in **Figure 3** will appear in front of you. To see a brief introduction about the server, please click on the “Read Me” button.
**Step 2**. Choose the one specie from *Saccharomyces cerevisiae* or *Pichia pastoris*.
**Step 3**. The input sequences should be in the FASTA format. The sequence data can be uploaded *via* the “Browse” button or copy and paste or type into the input box directly.
**Step 4**. To see the predicted results, please click on the “Submit” button. For example, if the four query DNA sequences in the Example window are used as the queried data, the predictive results are the 1^st^ and 2^nd^ query sequences are replication origins, and the 3^rd^ and 4^th^ are non-replication origins.

## Data Availability

Publicly available datasets were analyzed in this study. These data can be found here: https://academic.oup.com/bioinformatics/article-abstract/34/18/3086/4978052?redirectedFrom=fulltext

## Author Contributions

BL provided the main idea of the manuscript and wrote the manuscript. SC did the experiments and revised the manuscript. KY revised the manuscript and did the typesetting. FW did the experiments.

## Funding

This work was supported by the National Natural Science Foundation of China (No. 61672184, 61732012, 61822306), Fok Ying-Tung Education Foundation for Young Teachers in the Higher Education Institutions of China (161063), Shenzhen Overseas High Level Talents Innovation Foundation (Grant No. KQJSCX20170327161949608), Guangdong Natural Science Funds for Distinguished Young Scholars (2016A030306008), and Scientific Research Foundation in Shenzhen (JCYJ20180306172207178).

## Conflict of Interest Statement

The authors declare that the research was conducted in the absence of any commercial or financial relationships that could be construed as a potential conflict of interest.
