# Supplementary material for: iRO-PsekGCC: Identify DNA Replication Origins Based on Pseudo k-Tuple GC Composition
Source: Front Genet. 2019 Sep 18;10:842. doi: 10.3389/fgene.2019.00842 (PMC6759546; doi:10.3389/fgene.2019.00842)
Supplement: Supplementary file 1 [file Table_1.docx]

Supplementary Information S1

**Table S1.** The optimized parameters of the 10 basic RF classifiers, and their performance on the two benchmark datasets obtained by using 5-fold cross-validation

| **Species** | ***i*** | $\boldsymbol{\varepsilon}$ | $\boldsymbol{\delta}$ | $\boldsymbol{k}$ | $\boldsymbol{\lambda}$ | $\boldsymbol{w}$ | **ACC(%)** | $\boldsymbol{q}$ |
| --- | --- | --- | --- | --- | --- | --- | --- | --- |
| *Saccharomyces cerevisiae* $\mathbb{S}_{1}$ | 1 | 0.15 | 0.55 | 3 | 1 | 0.2 | 73.54 | 0.2011 |
|  | 2 | 0.15 | 0.55 | 3 | 3 | 0.2 | 71.93 | 0.0080 |
|  | 3 | 0.35 | 0.75 | 3 | 7 | 0.5 | 68.57 | 0.0877 |
|  | 4 | 0.35 | 0.85 | 3 | 1 | 0.7 | 73.98 | 0.0485 |
|  | 5 | 0.15 | 0.55 | 3 | 1 | 0.5 | 72.95 | 0.0387 |
|  | 6 | 0.15 | 0.55 | 3 | 1 | 0.1 | 73.10 | 0.0195 |
|  | 7 | 0.15 | 0.55 | 3 | 7 | 0.1 | 72.08 | 0.0504 |
|  | 8 | 0.35 | 0.85 | 3 | 7 | 0.3 | 72.66 | 0.2461 |
|  | 9 | 0.35 | 0.85 | 3 | 1 | 0.5 | 73.54 | 0.0326 |
|  | 10 | 0.35 | 0.55 | 3 | 7 | 0.1 | 73.54 | 0.2674 |
| *Pichia pastoris* $\mathbb{S}_{2}$ | 1 | 0.2 | 0.85 | 5 | 1 | 0.1 | 72.09 | 0.0013 |
|  | 2 | 0.2 | 0.85 | 5 | 1 | 0.3 | 71.92 | 0.2915 |
|  | 3 | 0.2 | 0.65 | 3 | 1 | 0.7 | 70.77 | 0.2082 |
|  | 4 | 0.4 | 0.8 | 6 | 4 | 0.1 | 68.97 | 0.0928 |
|  | 5 | 0.25 | 0.85 | 5 | 1 | 0.5 | 69.46 | 0.1272 |
|  | 6 | 0.3 | 0.8 | 4 | 1 | 0.1 | 70.11 | 0.0174 |
|  | 7 | 0.25 | 0.85 | 6 | 1 | 0.5 | 69.13 | 0.0072 |
|  | 8 | 0.35 | 0.85 | 6 | 1 | 0.3 | 70.77 | 0.2094 |
|  | 9 | 0.15 | 0.85 | 6 | 1 | 0.7 | 69.29 | 0.0379 |
|  | 10 | 0.2 | 0.85 | 5 | 4 | 0.1 | 71.43 | 0.0072 |
